# Methadone adverse reaction presenting with large increase in plasma methadone binding: a case series

**DOI:** 10.1186/1752-1947-5-513

**Published:** 2011-10-10

**Authors:** Wenjie J Lu, Weidong Zhou, Yvonne Kreutz, David A Flockhart

**Affiliations:** 1 Division of Clinical Pharmacology, Department of Pharmacology and Toxicology, Indiana University School of Medicine, Indianapolis, Indiana, USA; 2 Center for Applied Proteomics and Molecular Medicine, George Mason University, Manassas, Virginia, USA; 3 Division of Clinical Pharmacology, Department of Medicine, Indiana University School of Medicine, Indianapolis, Indiana, USA

## Abstract

**Introduction:**

The use of methadone as an analgesic is on the increase, but it is widely recognized that the goal of predictable and reproducible dosing is confounded by considerable variability in methadone pharmacokinetics, and unpredictable side effects that include sedation, respiratory depression and cardiac arrhythmias. The mechanisms underlying these unpredictable effects are frequently unclear. Here, to the best of our knowledge we present the first report of an association between accidental methadone overexposure and increased plasma protein binding, a new potential mechanism for drug interactions with methadone.

**Case presentation:**

We describe here the cases of two patients who experienced markedly different responses to the same dose of methadone during co-administration of letrozole. Both patients were post-menopausal Caucasian women who were among healthy volunteers participating in a clinical trial. Under the trial protocol both patients received 6 mg of intravenous methadone before and then after taking letrozole for seven days. One woman (aged 59) experienced symptoms consistent with opiate overexposure after the second dose of methadone that were reversed by naloxone, while the other (aged 49) did not. To understand the etiology of this event, we measured methadone pharmacokinetics in both patients. In our affected patient only, a fourfold to eightfold increase in methadone plasma concentrations after letrozole treatment was observed. Detailed pharmacokinetic analysis indicated no change in metabolism or renal elimination in our patient, but the percentage of unbound methadone in the plasma decreased 3.7-fold. As a result, the volume of distribution of methadone decreased approximately fourfold. The increased plasma binding in our affected patient was consistent with observed increases in plasma protein concentrations.

**Conclusions:**

The marked increase in the total plasma methadone concentration observed in our patient, and the enhanced pharmacodynamic effect, appear primarily due to a reduced volume of distribution. The extent of plasma methadone binding may help to explain the unpredictability of its pharmacokinetics. Changes in volume of distribution due to plasma binding may represent important causes of clinically meaningful drug interactions.

## Introduction

The use of methadone as an analgesic is on the increase [1], but it is widely recognized that the goal of predictable and reproducible dosing is confounded by considerable variability in methadone pharmacokinetics [2,3]. The unpredictability of methadone's effects results in a high incidence of inappropriate overdosing and underdosing, which can lead to severe adverse events including sedation, respiratory depression and cardiac arrhythmias [4-6]. Many such events, including death, occur beyond the reach of medical care or observation. As a result a complete understanding of the mechanisms underlying individual adverse events has rarely been possible. We present here a scenario in which we were able to carefully investigate the cause of an accidental methadone overexposure. To the best of our knowledge this is the first report of an association between accidental methadone overexposure and increased plasma protein binding.

## Case presentation

We describe here the cases of two patients who experienced markedly different responses to the same dose of methadone during co-administration of letrozole. Both patients were post-menopausal Caucasian women who were among healthy volunteers participating in a clinical trial (depicted in Table 1). The protocol for this study was approved by the Indiana University School of Medicine Institutional review Board (IRB), both patients signed informed consent before participation in the trial, and all procedures were conducted in accordance with the guidelines of the Declaration of Helsinki. The trial was designed to test the hypothesis that the aromatase inhibitor, letrozole, would alter the pharmacokinetics of methadone, based on pre-clinical data indicating that methadone is metabolized by aromatase *in vitro *[7]. Under the trial protocol both patients received 6 mg of intravenous methadone before, and then after taking letrozole for seven days.

**Table 1:** Clinical trial design and schedule of activities

	Screening	Period I				Washout period										Period II			
Study day	-28 to 0	01	02	03	04	05	06	07	08	09	10	11	12	13	14	15	16	17	18

Electrocardiogram for QT interval	X																		

Blood draw for screening	X																		

Methadone dose, 6.0 mg, intravenous		X														X			

Letrozole dose, 2.5 mg, orally once a day									X	X	X	X	X	X	X	X	X	X	X

Blood draw for pharmacokinetics		X	X	X	X											X	X	X	X

Urine sampling		X														X			

Blood samples (10 mL each) were collected before and at one, two, four, eight, 12, 24, 48 and 72 hours after each methadone dose, except in the second dose for our affected patient from whom blood samples at 48 and 72 hours could not be collected. Urine samples were collected at baseline and over the first 12 hours after methadone dose.

### Case 1

Our first patient was a 49-year-old Caucasian woman (69.5 kg, body mass index (BMI) 27.3, taking naproxen) who responded normally to the administered drugs as expected, and did not show any clinical adverse reaction to methadone or letrozole throughout the course of study. We designated this unaffected patient as 'N', and the doses of methadone administered before and after letrozole as dose 'A' and dose 'B' in the subsequent data analyses in order to compare the results between the two patients. All plasma and urine samples collected were analyzed as described in Additional file 1. No significant change in methadone pharmacokinetics before and after letrozole treatment was observed in our patient. Pharmacokinetic data obtained from patient 'N' were compared to those from Case 2 in details as described below.

### Case 2

**Figure 1:**
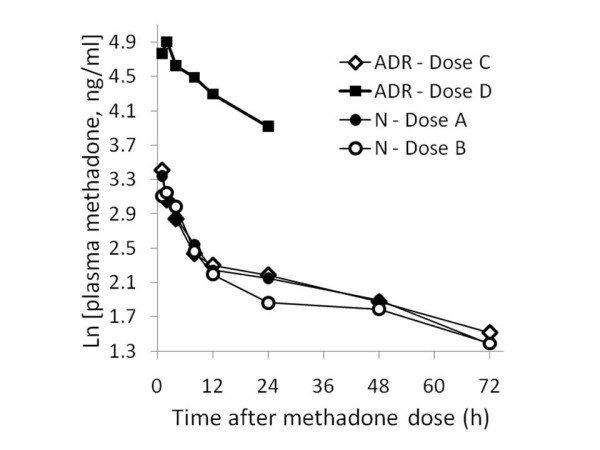
**Course of the adverse event over the first five hours after the dose of methadone**. The white arrows under the name of each medication indicate their times of administration. Shaded boxes on lines following specific symptoms indicate the times and duration of those symptoms. Double-headed arrow: during this time, vital signs worsened. The lowest blood pressure recorded was 110/86, respiratory rates as low as five breaths/minute occurred, and pulse oximetry documented oxygen saturation as low as 75%.

Our second patient was a 59-year-old Caucasian woman (86.8 kg, BMI 29.2, taking vitamin C, D, E, B6 and calcium). She experienced symptoms consistent with opiate overexposure that were reversed by naloxone after the second dose of methadone. Specific details of this adverse event, including the hospital course, patient monitoring data and medication schedule that resulted are illustrated in Figure 1. We designated our patient, who had experienced the adverse reaction, as 'ADR', and the doses of methadone administered before and after letrozole as dose 'C' and dose 'D' in the subsequent data analyses. Pharmacokinetic data from our ADR patient were compared in detail to those obtained from our N patient.

**Figure 2:**
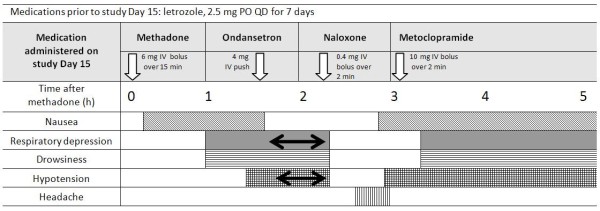
**Plasma methadone concentrations after single intravenous doses administered to both patients**. ADR = our patient who experienced methadone overexposure; doses A and C = doses of methadone administered before letrozole treatment; doses B and D = doses of methadone administered after letrozole treatment; N = our unaffected patient.

No methadone was detected in the plasma before the administration of intravenous methadone at baseline to either patient. Methadone plasma concentrations in ADR after dose D were fourfold to eightfold higher than those measured after her methadone dose C, given in the absence of letrozole (Figure 2). The maximum concentration observed was 135 ng/mL after dose D, while it was 30 ng/mL after dose C. When estimated pharmacokinetic parameters in our two patients were compared, the area under the curve (AUC)_0-24 h _of methadone, its redistribution half-life, and its volume of distribution (V_d_) were remarkably different after dose D in our ADR patient when compared to doses A, B and C (Table 2). These data indicate that there was a fourfold to sixfold decrease in V_d _in our ADR patient after dose D.

**Table 2:** Plasma pharmacokinetic parameters

Parameter	N			ADR		
	
	Dose A	Dose B	Ratio (B/A)	Dose C	Dose D	Ratio (D/C)
Letrozole C_baseline _(ng/mL)	0	106.9		0	76.1	

Methadone						

AUC_0-24 h _(ng/hour/mL)	288.5	272.2	0.94	291.2	1857	6.4

AUC_inf _(ng/hour/mL)	879.8	833.1	0.95	946.0	5674^a^	6.0

Distribution T_1/2 _(hour)	8.3	7.0	0.84	6.7	15.5	2.3

Elimination T_1/2 _(hour)	48.5	50.4	1.0	52.4	52.4^a^	1.0

Clearance (L/hour)	6.8	7.2	1.1	6.3	1.1^a^	0.17

V_d (extrap) _(L)	516.6	557.4	1.1	502.9	119.2^b^	0.24

V_d (area) _(L)	476.9	524.3	1.1	469.0	80.0^a^	0.17

EDDP						

AUC_0-24 h _(ng/hour/mL)	29.2	26.9	0.92	28.2	217.6	7.7

Observed C_max _(ng/mL)	1.6	1.3	0.81	1.6	10.5	6.6

AUC_methadone_/AUC_EDDP (0-12 h)_	12.3	13.6	1.1	12.6	11.6	0.92

AUC_methadone_/AUC_EDDP (0-24 h)_	9.9	10.1	1.0	10.3	8.5	0.83

EMDP						

AUC_0-24 h _(ng/hour/mL)	2.7	2.0	0.74	1.5	10.4	6.9

Observed C_max _(ng/mL)	0.18	0.14	0.78	0.075	0.55	7.3

^a^Estimated using clearance and half-life calculated from the terminal elimination phase.

^b^Estimated using an extrapolation method based on the terminal elimination phase.

AUC = area under the plasma concentration time curve; AUC_0-12 h _= AUC from time 0 to 12 hours; AUC_0-24 h _= AUC from time 0 to 24 hours; AUC_inf _= AUC from time 0 to infinity; ADR = our patient who experienced methadone overexposure; C = concentration; EDDP = 2-ethylidene-1,5-dimethyl-3,3-diphenylpyrrolidine (primary metabolite of methadone); EMDP = 2-ethyl-5-methyl-3,3-diphenyl-1-pyrroline (secondary metabolite of methadone); N = our unaffected patient; T_1/2 _= half-life; V_d _= volume of distribution.

The first 12-hour urine volume was much lower in ADR (dose D; Table 3). The concentration of urinary methadone after dose D was approximately 13-fold higher than that after dose C, and the total amount of methadone excreted was 1.4-fold greater.

**Table 3:** Urinary pharmacokinetic parameters

Parameter	N			ADR		
	
	Dose A	Dose B	Ratio (B/A)	Dose C	Dose D	Ratio (D/C)
Total urine volume (12 h, mL)	3132	3090	0.99	2488	267	0.11

Methadone						

C (ng/mL)	52.6	54.6	1.0	88.6	1168	13.2

Total mass (μg)	165	169	1.0	220	312	1.4

Mass_total_/AUC_0-12 h _(L/hour)	0.84	0.88	1.0	1.14	0.26	0.23

EDDP						

C (ng/mL)	12.7	8.97	0.71	25.5	424	16.6

Total mass (μg)	39.7	27.7	0.70	63.4	113	1.8

C_(methadone)_/C_(EDDP)_	4.14	6.09	1.47	3.47	2.75	0.79

EMDP						

C (ng/mL)	0.044	0.036	0.82	0.019	0.23	12.1

Total mass (μg)	0.14	0.11	0.79	0.047	0.062	1.3

AUC = area under the plasma concentration time curve; AUC_0-12 h _= AUC from time 0 to 12 hours; ADR = our patient who experienced methadone overexposure; C = concentration; EDDP = 2-ethylidene-1,5-dimethyl-3,3-diphenylpyrrolidine (primary metabolite of methadone); EMDP = 2-ethyl-5-methyl-3,3-diphenyl-1-pyrroline (secondary metabolite of methadone); N = our unaffected patient.

The metabolite data from our ADR patient also indicate important differences after dose D, both in the plasma (Table 2) and in the urine (Table 3). The AUC_0-24 h _of plasma 2-ethylidene-1,5-dimethyl-3,3-diphenylpyrrolidine (EDDP; primary metabolite of methadone) and 2-ethyl-5-methyl-3,3-diphenyl-1-pyrroline (EMDP; secondary metabolite of methadone) increased approximately eightfold and approximately sevenfold respectively. The urinary concentration of EDDP increased approximately 17-fold, and the concentration of EMDP increased approximately 12-fold. The total amounts of EDDP and EMDP excreted in urine during the first 12 hours were 1.8-fold and 1.3-fold greater respectively than those after dose C (Table 3).

In order to determine the amount of bound and free methadone and EDDP in the plasma, we selected samples collected at eight and 12 hours after the methadone dose, which is after the redistribution phase assuming a two-compartment model. In our ADR patient, the mean methadone fraction unbound (*f_u_*) in these samples decreased 3.7-fold from doses C to D, while the EDDP fraction unbound decreased 3.6-fold (Table 4).

**Table 4:** Pharmacokinetic indices for plasma drug binding at eight and 12 hours after methadone dosing

Parameter	N			ADR		
	
	Dose A		Dose B	Dose C		Dose D
Methadone						

C_free, 8 h _(ng/mL)	1.40		1.43	1.36		3.18

C_bound, 8 h _(ng/mL)	8.45		8.22	8.72		78.0

C_free, 12 h _(ng/mL)	1.18		1.10	1.38		2.61

C_bound, 12 h _(ng/mL)	6.86		6.74	7.40		58.8

Mean *f_u_*	0.14		0.14	0.15		0.04

*f_u _*ratio (A/B or C/D)		1.0			3.7	

V_d (extrap) _ratio		0.93			4.2	

V_d (area) _ratio		0.91			5.9	

EDDP						

C_free, 8 h _(ng/mL)	0.38		0.39	0.35		0.75

C_bound, 8 h _(ng/mL)	0.72		0.75	0.61		6.56

C_free, 12 h _(ng/mL)	0.36		0.36	0.38		0.77

C_bound, 12 h _(ng/mL)	0.71		0.66	0.69		7.37

Mean *f_u_*	0.34		0.35	0.36		0.10

*f_u _*ratio (A/B or C/D)		1.0			3.6	

ADR = our patient who experienced methadone overexposure; C = concentration; EDDP = 2-ethylidene-1,5-dimethyl-3,3-diphenylpyrrolidine (primary metabolite of methadone); *f_u _*= fraction unbound; N = our unaffected patient.

When plasma protein concentrations were measured in these samples, an increase in total protein of 0.8 to 1.2 g/dL (13% to 20%) was observed after dose D (Table 5). Upon further analysis of the whole plasma proteome by MS and relative quantification on the basis of label-free spectra count, proteins that appeared to increase by twofold to fourfold during the adverse event (dose D) relative to samples drawn at the equivalent time before (dose C) included thrombin and its precursors, fibrinogen and its precursors, complement factor 1, retinol-binding protein 4, Shwachman-Bodian-Diamond syndrome protein, apolipoprotein A-IV, serpin peptidase inhibitor, clade C and kininogen 1 isoform 2 (data not shown).

**Table 5:** Plasma protein concentrations and serum chemistries at eight and 12 hours after methadone dosing

Parameter	N		ADR	
	
	Dose A	Dose B	Dose C	Dose D
Plasma proteins				

Total protein_8 h _(g/dL)	5.8	5.8	5.9	7.1

Albumin_8 h _(g/dL)	3.4	3.5	3.4	3.8

AAG_8 h _(mg/dL)	84	86	74	87

Total protein_12 h _(g/dL)	6.3	6.0	6.1	6.9

Albumin_12 h _(g/dL)	3.5	3.5	3.6	3.8

AAG_12 h _(mg/dL)	79	81	81	85

Serum chemistries				

Na^+ ^(mmol/L)			144	139

Cl^- ^(mmol/L)			108	104

HCO_3_^- ^(mmol/L)			29	25

Blood urea nitrogen (mg/dL)			15	15

Creatinine (mg/dL)			0.78	0.72

AAG = α_1_-acid glycoprotein; ADR = our patient who experienced methadone overexposure; N = our unaffected patient.

In addition, serum concentrations of Na^+^, Cl^-^, HCO_3_^-^, blood urea nitrogen and creatinine in our ADR patient were similar after both doses C and D (Table 5). Serum lipid profiles were also similar before and after dosing (data not shown).

## Discussion

No interaction between methadone and letrozole had been previously described. As a result of this adverse event, the investigators immediately informed the IRB, and put the trial on hold so that the cause of this adverse event could be carefully investigated before any adjustments were made to the trial design.

Unanticipated adverse events and fatalities caused by methadone are a significant public health problem [4-6]. In this case report, we observed a marked increase in plasma methadone concentrations and symptomatic overexposure during co-administration of letrozole to a single patient. Pharmacokinetic sampling was limited to the first 24 hours after methadone dosing during the adverse event, but large pharmacokinetic changes were obvious. Since such large intra-individual changes may have occurred in other patients, we estimated pharmacokinetic parameters in order to explore the multiple possible causes of this adverse reaction.

First, although the data appear similar to those that might be obtained as the result of a dosing error, the amount of methadone remaining (single vial) before and after this event were measured, and showed that the correct dose was used. The measured decrease in plasma *f_u _*also makes an overdose seem impossible.

Second, the increase in methadone exposure was not due to decreased metabolism, since the parent to metabolite ratio in both plasma and urine decreased in our patient.

Third, although the observed renal clearance of methadone significantly decreased (Table 3), reduced urinary clearance was not the cause of the pharmacokinetic changes observed in our patient since the total urinary drug excreted over 12 hours was higher rather than lower (Table 3).

Fourth, a small decrease in plasma volume could contribute to the total increase in drug concentrations, but serum electrolyte concentrations were similar after both methadone doses, suggesting our patient was not substantially hypovolemic. A small decrease in plasma volume cannot explain such large pharmacokinetic changes.

Fifth, a change in transporter activity could in theory increase plasma concentrations. The transport system involved would have to counteract the rapid tissue diffusion of methadone, a lipophilic drug, by stimulating active transport of drug back into the plasma. While this is theoretically possible, methadone has not been shown to be vulnerable to transporter-mediated interactions, and no effect of letrozole on drug transporters has ever been described previously.

Sixth, we did observe a greater than fourfold decrease in the estimated V_d _(Table 4), which could result in increased plasma and urine concentrations. V_d _can be described using the following equation:

$${\text{V}}_{\text{d}}\text{ = ECF + }\Phi ∙f_{u}∙\text{ICF}$$

where ECF is the volume of the extra-cellular fluid, Φ is the tissue-binding factor, *f_u _*is the plasma fraction unbound, and ICF is the volume of the intra-cellular fluid [8]. Relative to the terms in the second half of the equation, ECF is a tiny contributor to the total V_d_, especially for a drug with a large V_d _such as methadone [9]. A decrease in tissue binding would not be expected to reduce the *f_u _*in plasma, and cannot explain the increase in plasma binding actually observed. In contrast, we observed a decrease in *f_u _*due to increased plasma binding of methadone and its metabolites. A 3.7-fold change in *f_u _*is of similar scale to that in V_d_, and therefore can account for most of the change. This change was also accompanied by an increase in free methadone concentration from 1.36 to 3.18 ng/mL in our affected patient. The concentration of bound methadone increased from 8.72 to 78.0 ng/mL. While it is clear that the increased concentration of unbound methadone contributed to this symptomatic overexposure, the large change in bound methadone may also have contributed by making available a large reservoir of free drug for transport or diffusion across the blood brain barrier. In addition, we observed an increase in plasma protein concentrations, and this is consistent with the increase in plasma methadone binding. Of note, increased plasma protein concentration has been previously reported to result in decreased methadone *f_u _*[10].

Methadone has been shown to bind to a number of different plasma proteins [11,12], including α_1_-acid glycoprotein (AAG), β-globulin [11] and lipoprotein fractions [12]. When we examined changes in the plasma proteome after dose D relative to dose C, we noted small increases in the concentrations of albumin and AAG, but prominent approximately twofold increases in proteins in the coagulation pathway: in concentrations of thrombin and its precursors, fibrinogen and its precursors and complement factor 1. Which of these might be responsible for the change in methadone binding observed in our patient is at present unclear, but the suggestion that changes in the coagulation pathway may result in altered drug binding is an observation that may have significance in some clinical scenarios.

The observed change in binding could not have been due to the ingestion of other drugs, since neither of the women were taking any medicine known to alter methadone binding. It is possible that letrozole caused these changes in plasma protein concentrations via its effect on estrogen concentrations [13,14]. While few data are available on such effects of estrogen depletion, it is clear that estrogen supplementation with hormone replacement therapy can decrease the concentrations of AAG [15]. It follows that AAG may increase during therapy with an aromatase inhibitor, as was observed in this case. It is also possible that letrozole brings about these changes via an 'off-target' effect, or that the change we observed in methadone plasma binding is not due to letrozole, but due to some uncontrolled factor.

## Conclusions

This study illustrates a novel mechanism underlying intra-individual changes in methadone pharmacokinetics and pharmacodynamics: increases in plasma binding that could result in increased effects. This mechanism might help explain the unpredictability of methadone effects. A similar mechanism may be responsible for interactions with other drugs that alter the concentrations of plasma binding proteins. The consequent changes in V_d _may represent important causes of clinically meaningful drug interactions.

## Consent

Written informed consent was obtained from both patients for publication of this case report and any accompanying images. Copies of the written consents are available for review by the Editor-in-Chief of this journal.

## Competing interests

The authors declare that they have no competing interests.

## Authors' contributions

WL and DF participated in the design and conduct of the clinical trial, and are the major contributors in writing the manuscript. WL analyzed and interpreted the pharmacokinetic data on methadone and its metabolites. WZ carried out proteomic analysis on plasma samples. YK analyzed letrozole concentrations from plasma samples. All authors read and approved the final manuscript.

## Supplementary Material

Additional file 1 **Methods and materials**. Supplementary materials and methods. Click here for file
