# Supplementary material for: Methadone adverse reaction presenting with large increase in plasma methadone binding: a case series
Source: J Med Case Rep. 2011 Oct 10;5:513. doi: 10.1186/1752-1947-5-513 (PMC3201932; doi:10.1186/1752-1947-5-513)
Supplement: Additional file 1 — Methods and materials. Supplementary materials and methods. [file 1752-1947-5-513-S1.DOC]

**Materials and Methods**

**Chemicals and Reagents**

Methadone for IV administration was obtained from Xanodyne (Newport, KY) as a 10 mg/ml solution.  Volumes of methadone used for each administration were recorded as 0.6 ml (6 mg). This dose was diluted in 50 ml of normal saline for IV infusion. The same lot number was used for each administration of methadone, and was prepared by the Indiana University Hospital Pharmacy, Investigational Drug Services. Letrozole pills (Femara® 2.5 mg tablets) were obtained from Novartis (Basel, Switzerland).

Racemic methadone hydrochloride and desmethyldiazepam were purchased from Sigma-Aldrich (St. Louis, MO). 2-ethylidene-1,5-dimethyl-3,3-diphenylpyrrolidine (EDDP) iodide and 2-ethyl-5-methyl-3,3-diphenylpyrroline (EMDP) hydrochloride were obtained from Alltech (Deerfield, IL). Nevirapine and letrozole were purchased from Toronto Research Chemicals Inc. (North York, ON, Canada). All HPLC-grade reagents and chemicals used for mobile phase and buffers were obtained as previously described^8^. Methyl tert-butyl ether was purchased from Fisher Scientific (Pittsburgh, PA). All solutions of methadone, EDDP, EMDP, letrozole, nevirapine, desmethyldiazepam were prepared by dissolution in methanol, and were stored at −20 °C.

**Subjects**

Subjects enrolled in this clinical trial were healthy post-menopausal women 45 to 70 years of age who weighed at least 110 lbs, and who agreed to avoid the use of alcohol during the period of the study. Potential subjects were excluded from the study if they had a history of intolerance or allergy to methadone or letrozole, had a diagnosis of asthma, chronic obstructive pulmonary disease, cor pulmonale, severe obesity with a weight over 300 lbs, or sleep apnea syndrome. Subjects were also excluded if they were anemic (hematocrit below 30%), suffered from renal insufficiency (serum creatinine greater than 1.4) or had elevation of serum liver enzymes, had low serum potassium, a prolonged electrocardiographic QTc interval of more than 480 msecs (Bazette), or were taking drugs know to prolong the QT interval. The protocol for this study was approved by the Indiana University School of Medicine IRB, all subjects signed informed consent before participation in the trial, and all procedures were conducted in accordance with the guidelines of the Declaration of Helsinki.

**Sample Analysis**

Plasma letrozole concentrations were measured using high performance liquid chromatography (HPLC) assays with ultraviolet and fluorescence detection as described below. Baseline plasma samples (200 μl) were made alkaline by adding 500 µL of 1 M glycine-NaOH buffer (pH 11.3) and extracted by adding 6 mL of ethyl acetate. Desmethyldiazepam (100 μl of 1000 ng/ml) was used as internal standard. This mixture was mixed on a reciprocal shaker **(Eberbach, Ann Arbor, MI)** for 5 min and then centrifuged at about 3,000 rcf for 15 min. The organic layer was transferred to 13×100-mm glass culture tubes and evaporated to dryness. The resulting residue was reconstituted with mobile phase (70% 10 mM monobasic potassium phosphate, 30% acetonitrile, pH adjusted to 6.5) and analyzed immediately. The HPLC system was controlled by CLASS-VP version 7.1.1 SP1 Chromatographic Software (Shimadzu Scientific Instruments Inc., Columbia, MD) and included a Shimadzu solvent delivery module SCL-10A VP, an autoinjector SIL-10AD VP, a spectrofluorometric detector RF-10A XL (set at an excitation wavelength of 230 nm and emission wavelength of 295 nm), an ultraviolet detector SPD-10A VP (set at a wavelength of 234 nm, 0.1 AUFS) and a system controller SCL-10A VP (Shimadzu, Kyoto, Japan). The eluate was directed at 1 ml/min through a Zorbax SB-C18 3.5-μm C_18_ column (150 x 4.6 mm i.d.; Agilent Technologies, Santa Clara, CA) coupled with a Nova-Pak C_18_ Guard column (4 μm; Waters, Inc., Ireland) with a run time of 32 min. Under these conditions, the retention times of letrozole and desmethyldiazepam were approximately 17 and 27 min, respectively.

HPLC with tandem mass spectrometry detection (HPLC-MS/MS) assays were developed for the quantification of methadone and its major metabolites, EDDP and EMDP. Each plasma and urine sample (1 ml) was extracted by adding 500µL of 1M glycine-HCl buffer (pH 3.0) and extracted by adding 6 mL of methyl tert-butyl ether. Nevirapine (100 µl of 1 µg/ml) was added to each sample as an internal standard. All samples were mixed, centrifuged and transferred as described above, and then reconstituted with mobile phase (50% 20 mM ammonium acetate and 50% acetonitrile without pH adjustment) and analyzed immediately. The HPLC-MS/MS assay was performed on an Applied Biosystems (Foster City, CA) model API 2000 triple-quadropole mass spectrometer, coupled with a Shimadzu (Addison, IL) HPLC system consisting of a model LC-20AB binary solvent delivery pump and model SIL-20A HT autosampler. The separation system was composed of a Luna 3 μm C_18_-2 column (100 x 2.00 mm i.d.; Phenomenex, Torrance, CA) and a nitrile guard column (4 x 3.0 mm; Phenomenex). The eluate was introduced, without splitting, at 0.200 mL/min to the electrospray ionization source. The electrospray voltage was set at +2500 mV and the dwell time at 100 ms per detection channel with unit mass resolution on the quadrupole 1 and 3 mass analyzers. Optimal gas pressures for all of the analytes were: nitrogen nebulizer gas 20.00 psi, turbo/heater gas 0.00 psi, curtain gas 20.00 psi and collision gas 2.00 psi. All data were collected in the positive ion mode with the temperature of the interface set at 450°C. Under these conditions, nevirapine, methadone, EDDP and EMDP were detected at the retention times of approximately 3, 6, 5, and 10 min, respectively, and were quantified using m/z values (Q3/Q1) at 226.0/267, 265.2/310, 234.1/278 and 220.2/264 respectively.

Peak areas for each peak were obtained, and peak area ratios with internal standard were calculated. Standard curves were constructed by linear regression of peak area ratios. Quantification of samples was carried out by applying the linear regression equation of the standard curve to the peak area ratio. Using these methods, the limits of quantification (LOQ) for methadone, EDDP, EMDP and letrozole were 0.1, 0.2, 0.04 and 12.5 ng/ml respectively with intra-day and inter-day coefficients of variation of less than 4.3% and less than 8.1% respectively, when measured above the LOQs.

The extent of plasma binding of methadone and its metabolites was determined using ultrafiltration. Plasma samples (500 µl each) were processed (room temperature) through Microcon® Ultracel YM-3 (Millipore, Billerica, MA) centrifugal filters. Filtrate and retentate drugs were measured as described above.

Plasma total protein, albumin and α_1_-acid-glycoprotein concentrations and serum chemistries were measured by the Indiana University School of Medicine Department of Pathology clinical laboratory. Analyses of the plasma proteome were carried out in the laboratory of Lance Liotta PhD at George Mason University using methods described in detail elsewhere^9^.

**Determination of pharmacokinetic parameters**

Data on methadone and its metabolites including concentration-time curve (AUC), half life (T_½_), plasma clearance (CL) and volume of distribution (V_d_) were estimated using a two-compartment model (Phoenix™ WinNonlin® 6.1, Pharsight Corporation, Cary, NC). Two approaches were applied to estimate V_d_. First, V_d_ was calculated based on the terminal elimination phase as V_d_ _(area)_ using the following equation:

V_d (area)_ = CL · T_½_ / 0.693

Since an accurate determination of T_½_ could not be obtained due to the brevity of the sampling time after the adverse reaction, we employed the conservative assumption that there was no change in the T_½_ of methadone. Since the data actually suggest a faster rate of metabolism and no decrease in renal elimination (see Results), this assumption is most likely to result in an overestimate of the V_d_, and underestimate of its contribution to the overall pharmacokinetic change. Second, we calculated V_d_ using the following equation:

V_d (extrap)_ = Dose/ C_0_

where C_0_ is the extrapolated initial plasma methadone concentration at the time of dosing, assuming complete distribution. In order to minimize the risk of overestimating C_0_ during the adverse event, and to obtain a conservative estimate of this change in V_d_, we estimated C_0_ for this dose by simple extrapolation of a horizontal line drawn through the 24 hr data point.
